# Why Controlling Teaching Persists in Medical Education: A Self-Determination Theory Perspective

**DOI:** 10.5334/pme.2417

**Published:** 2026-09-24

**Authors:** Adam Neufeld

**Affiliations:** 1 Department of Family Medicine, Cumming School of Medicine, University of Calgary, Calgary, Alberta, Canada

## Abstract

Autonomy-supportive teaching is widely promoted in medical education for its benefits to learner motivation and well-being. Yet many learners continue to experience clinical education as controlling, even in environments that explicitly endorse learner-centred values. This persistent gap suggests that barriers to autonomy-supportive practice extend beyond lack of knowledge or skill. This paper offers a theory-informed analysis of why psychologically controlling teaching persists in high-stakes clinical learning environments. Using Self-Determination Theory (SDT) as its organizing framework and drawing on complementary perspectives from psychological reactance, stress and coping, motivating styles, and medical education systems, the paper advances four interconnected arguments. Controlling teaching may emerge when educators experience pressure, threatened agency, responsibility, and uncertainty; be reinforced when authority and instructional structure are mistaken for psychological control; be legitimized through professional norms that associate control with competence; and be reproduced through assessment, administrative, and curricular systems. Its persistence is understandable because controlling approaches often produce the immediate outcomes educators seek—compliance, efficiency, predictability, and reduced uncertainty—even while carrying longer-term motivational costs. Control is therefore conceptualized not simply as an individual pedagogical failure, but as a downstream behavioural expression of pressure travelling through educational systems. By making these mechanisms visible, the paper provides a conceptual foundation for developing autonomy-supportive practices that remain viable under pressure and offers practical implications for educators, faculty development, and educational systems.

## Introduction

Self-Determination Theory (SDT) proposes that social environments influence motivation and well-being partly through their support or frustration of three basic psychological needs: autonomy, competence, and relatedness [1]. Autonomy concerns experiencing one’s actions as volitional and personally endorsed; competence concerns feeling effective and capable of growth; and relatedness concerns feeling respected, connected, and significant to others. Need-supportive environments facilitate autonomous motivation and the internalization of values and responsibilities, whereas need-frustrating environments organize behaviour around pressure, avoidance, defensiveness, and disengagement. These principles have informed a growing body of medical education scholarship demonstrating the benefits of autonomy-supportive teaching and identifying practical strategies for supporting learners’ motivation, development, and well-being [2,3,4].

Yet many learners continue to experience clinical education as pressuring, evaluative, or controlling, even in settings where educators explicitly endorse learner-centred values [5,6,7]. This gap between espoused values and lived experience suggests that barriers to autonomy-supportive practice extend beyond knowledge deficits or skill alone. Even well-designed interventions may fail to translate into sustained practice when contextual pressures, institutional demands, and educators’ own psychological needs remain unaddressed [8,9].

The present paper therefore shifts attention from autonomy support to its often-overlooked counterpart: psychologically controlling teaching. Throughout this paper, “control” refers to interpersonal behaviours that pressure learners to think, feel, or behave in particular ways through coercion, surveillance, conditional regard, guilt, intimidation, or other forms of external or internal pressure. It does not refer to clear expectations, instructional structure, legitimate clinical authority, supervision, decisiveness, or direct intervention when patient safety is at risk. These may all be enacted in autonomy-supportive ways when learners’ perspectives are acknowledged, expectations are explained, and authority is exercised without unnecessary psychological pressure.

Rather than reiterating what autonomy-supportive teaching is or how to enact it—topics addressed extensively elsewhere [10,11,12,13]—this paper asks why controlling teaching persists so reliably across undergraduate and postgraduate medical education, even among educators who value learner-centredness and understand autonomy-supportive principles. Recent SDT research shows that autonomy-supportive and controlling approaches are not simply opposite ends of a continuum, but partially independent motivating styles that predict distinct motivational pathways. Autonomy support predicts psychological need satisfaction and adaptive functioning, whereas control uniquely predicts psychological need frustration and maladaptive outcomes [14,15,16]. Reducing controlling teaching therefore requires more than increasing autonomy support.

This distinction also directs attention toward educators’ own motivational experiences. Educators do not enter teaching encounters untouched by the systems around them: they may themselves feel constrained by assessment requirements, curriculum demands, clinical workload, institutional expectations, patient-safety responsibilities, or learners who appear disengaged. Research outside medical education has shown that pressure from “above,” including curriculum and performance demands, and pressure from “below,” including perceptions that students are poorly motivated, can diminish teachers’ autonomous motivation and indirectly increase their use of controlling teaching practices [17].

Together, these findings suggest that pressure rarely remains where it originates. Institutional demands shape educators’ motivational experiences, those experiences influence how authority is exercised, and learners ultimately experience the downstream consequences. In this way, pressure can travel through educational systems. Psychological reactance offers one complementary explanation for how this translation occurs. Threats to perceived freedom can generate a motivational impulse to restore agency through resistance, assertion, or efforts to regain control [18]. Reactance does not imply that every constrained educator will become controlling, but it helps explain why feeling controlled can increase the psychological appeal of controlling others.

This paper develops four interconnected arguments. First, controlling teaching can arise as educators respond to pressure, threatened agency, responsibility, and uncertainty. Second, it is reinforced when autonomy support is confused with permissiveness and psychological control with necessary structure or authority. Third, professional cultures may legitimize control by associating decisiveness, certainty, and dominance with competence. Fourth, assessment, curricular, and administrative systems can reproduce control even when their underlying educational structures are sound. Rather than locating control primarily within individual educators, the resulting framework conceptualizes it as an emergent property of interactions among educators, learners, professional cultures, and educational systems. Its central claim is that controlling teaching is often the downstream behavioural expression of pressure travelling through those systems.

## Pressure Travels Downward: Control as a Response to Threatened Agency and Responsibility

Within SDT, teaching and supervisory approaches are understood as motivating styles: the interpersonal tone and behaviours educators use to engage learners. Autonomy-supportive educators acknowledge learners’ perspectives, provide meaningful rationales, offer choice within appropriate structure, and support volitional engagement [19,20,21]. Controlling approaches instead rely on pressure, surveillance, conditional regard, intimidation, guilt, or compliance-inducing language, contributing to psychological need frustration [22].

Controlling teaching is rarely driven by poor intentions or professional disregard. Educators’ motivating styles are shaped by what they perceive to be effective, professionally responsible, and feasible within their environments [23,24]. Pelletier and colleagues demonstrated this empirically: teachers who perceived greater pressure from institutional requirements and performance standards, as well as pressure arising from students they perceived as poorly motivated, reported less autonomous motivation toward teaching and, in turn, adopted more controlling teaching behaviours [17]. Pressure imposed on educators was therefore carried forward into their interactions with learners.

Clinical educators experience comparable pressures from multiple directions. Our recent BEME systematic review synthesized these as influences from above (organizational and societal expectations), within (educators’ beliefs, dispositions, and reasons for teaching), and below (perceptions of learners’ engagement and motivation) [25]. Although the review focused on motivation to teach rather than controlling behaviour, it demonstrated that educators’ motivation is embedded within a multilevel social system. Pressure therefore rarely originates from a single source; it accumulates across roles, relationships, and institutional expectations before being expressed in everyday teaching. Clinical educators may simultaneously be expected to meet curricular requirements, maintain productivity, ensure patient safety, assess learners, manage uncertainty, and engage trainees who vary in readiness and motivation. These accumulated demands shape not only whether clinicians are motivated to teach, but also how they exercise authority when they do.

Motivating styles are also shaped by relatively stable individual differences. Educators higher in control causality orientation are more likely to perceive their environments as evaluative and demanding, increasing the likelihood that uncertainty is met with psychologically controlling responses, particularly when accompanied by authoritarian beliefs or low tolerance for ambiguity [26]. These tendencies are especially relevant in medicine, where responsibility, accountability, and uncertainty are enduring features of clinical work. The same situation may therefore invite one educator to guide, while prompting another to restore control, depending on how external demands are interpreted and internalized.

Psychological reactance further clarifies how threatened agency may be translated into controlling behaviour. Reactance is the motivational state directed toward restoring freedom following its perceived restriction or loss [18]. It may be expressed through resistance, anger, assertion, derogation of the source of pressure, or attempts to re-establish control. In clinical education, educators constrained by time, policy, assessment requirements, or institutional surveillance often have limited ability to alter the source of those pressures. Reasserting control within the teaching relationship can therefore become one of the few remaining ways to restore agency, predictability, or professional authority. This does not make controlling teaching inevitable, nor does it imply that frustration is consciously displaced onto learners. It does, however, help explain why feeling controlled can increase the psychological appeal of becoming more controlling.

Stress and coping theory adds a further layer. When educators experience anxiety related to learner assessment, patient safety, clinical uncertainty, or external evaluation, controlling behaviours may provide short-term relief by narrowing choice and increasing predictability [27]. Reactance describes the impulse to regain threatened agency, coping theory describes attempts to regulate the resulting distress and uncertainty, and SDT explains how both are shaped by social conditions that support or frustrate psychological needs. Viewed together, controlling teaching can regulate both the external situation and the educator’s internal state.

Control may therefore become psychologically self-reinforcing. It relieves anxiety, restores a sense of agency, and produces immediate compliance, increasing the likelihood that educators will rely on it again when similar demands arise. This helps explain why controlling teaching can persist despite learner-centred values: its immediate benefits are experienced by educators, whereas many of its motivational costs are delayed, less visible, and borne primarily by learners.

Pressure alone, however, does not determine how educators respond. Its translation into controlling teaching is also shaped by what educators believe authority, guidance, and effective supervision should look like.

## When Authority and Structure are Confused with Psychological Control

Autonomy support is often misinterpreted as permissiveness, reduced standards, excessive learner choice, or a lack of direction. Prior work has addressed this misunderstanding by distinguishing autonomy from independence: autonomy concerns learners’ experience of volition and ownership, not freedom from guidance, supervision, accountability, or clinical authority [28]. When responsibility is acute, failure to distinguish these constructs can lead educators to view psychological pressure as necessary for maintaining standards or protecting patients.

Empirical evidence consistently shows that autonomy support and structure are complementary rather than competing features of effective teaching. For example, a recent experimental vignette study among junior doctors found that autonomy-supportive supervision predicted greater psychological need satisfaction and intrinsic motivation, with these benefits strengthened rather than diminished when supervisors also provided clear direction [29]. The relevant distinction is therefore not between authority and autonomy support, but between authority enacted through clear, informational, and need-supportive guidance and authority enacted through unnecessary psychological pressure.

Perhaps the best analogy is a jungle gym rather than an open field. A jungle gym provides clear boundaries, scaffolding, and safety features, while still allowing learners to move freely, experiment, and take initiative. In educational terms, autonomy support does not mean removing structure; it means designing structure so learners can act with agency within clear expectations. Decision latitude, responsibility, and challenge are calibrated according to level of training—not as “graduated autonomy,” but as graduated responsibility and independence delivered in a need-supportive way. Such structure helps learners feel safe, oriented, and capable while reducing the perceived need for educators to manage uncertainty by constraining others [30,31].

Research examining combinations of perceived autonomy support and control reinforces this distinction. The most adaptive motivational profile is characterized by high autonomy support and low control, whereas high control predicts need frustration and maladaptive outcomes even when autonomy support is also present [16]. Pressure is therefore not a neutral supplement to structure—it carries distinct motivational costs. The challenge is not to make clinical teaching less directive, but to distinguish direction that develops competence from pressure that just secures compliance.

Whether educators make this distinction is influenced by the cultures in which they learned what authority and competence look like. Practices that appear controlling from an SDT perspective may be interpreted within medicine as signs of confidence, rigour, decisiveness, or professional responsibility.

## Why Professional Cultures Legitimize Control

Medical training has long privileged hierarchy, authority, and certainty—traditions shaped by historical models of expertise that centralized knowledge, rewarded decisiveness, and discouraged deviation [32,33]. Although contemporary medicine has made meaningful strides toward collaboration and inclusivity, many of these assumptions persist in subtle and often unquestioned forms. Educational cultures may therefore continue to reward decisiveness over deliberation, compliance over curiosity, and emotional restraint over reflection and integration, reinforcing implicit beliefs about what competent teachers should look like.

Norms surrounding professionalism and competence can consequently come to valorize certainty, endurance, and emotional suppression over dialogue, reflection, and shared inquiry [34,35]. Malin and Palmer’s analysis of “pimping” illustrates how fear-based teaching may produce strong motivation, but primarily of a controlled kind that undermines internalization, psychological safety, and well-being [36]. Within such cultures, psychologically controlling teaching may come to be viewed not as a regrettable compromise, but as an expected expression of competence, authority, and professional responsibility.

This process is reinforced through how competence itself is perceived and evaluated. One mechanism through which this occurs is a perceptual bias: controlling educators may be viewed as more competent than their less controlling counterparts. In a foundational study, controlling instructional directives led to poorer learner performance, yet the controlling teacher was nevertheless rated as more competent than a non-controlling teacher using the same instructional strategy [37]. In high-stakes, analytically oriented professions such as medicine, where confidence and decisiveness are highly valued, this bias may further normalize control by making it appear both effective and professionally desirable.

Control is therefore sustained not only because it produces short-term compliance, but because medical culture may reward its appearance. Learners and educators are socialized within environments where certainty can be mistaken for expertise, pressure for rigour, and obedience for professionalism. These interpretations make controlling practices easier to reproduce and harder to recognize as modifiable.

Professional culture does not operate only through interpersonal norms. It is also translated into assessment requirements, administrative communications, curricular processes, and educational technologies that organize learners’ everyday experience.

## How Educational Systems Reproduce Control

Administrative communications, assessment processes, curricular policies, and program procedures communicate implicit expectations about authority, accountability, and acceptable behaviour. These interactions may be experienced as unilateral, compliance-focused, and stripped of meaningful rationale, despite often being developed with good intentions. Emails, policies, and syllabi may adopt commanding or legalistic language, invoke professionalism as a broad but enforceable standard, and provide limited opportunity for dialogue or contextual understanding. Because these interactions carry evaluative and disciplinary weight, learners may come to perceive questioning decisions, requesting flexibility, or expressing disagreement as personally or professionally risky.

Research in medical education demonstrates that assessment systems and policy environments shape learner behaviour through fear of evaluation, impression management, and perceived risk, even when no individual educator intends harm [38,39]. Recent qualitative work examining residents’ experiences of Entrustable Professional Activities (EPAs) illustrates this process particularly well. Although EPAs were designed to support workplace learning and professional development, residents frequently experienced them as documentation exercises driven by compliance rather than learning. Documentation requirements constrained autonomy, fragmented competence development, and weakened meaningful educational dialogue, while electronic systems often replaced formative conversations with transactional interactions [40].

These findings do not suggest that EPAs or competency-based medical education are inherently controlling. Rather, they demonstrate that sound educational structures can become need-thwarting through the way they are framed, operationalized, and experienced. Structures intended to support development may instead produce pressure when documentation displaces dialogue, accumulation replaces reflection, and assessment becomes disconnected from meaningful clinical work. The relevant issue is therefore not simply which structures medical education adopts, but how those structures are enacted.

Control becomes embedded not only in interpersonal teaching behaviours, but also in the organizational conditions surrounding them. Over time, these upstream influences externalize motivational costs onto learners and render control less visible as a modifiable feature of educational design. In this way, pressure travels through the system: institutional demands shape educator motivation and behaviour, cultural norms legitimize controlling responses, and educational structures transmit those responses to learners. Control persists not simply because educators choose it, but because the surrounding system repeatedly makes it appear appropriate, efficient, and professionally necessary.

## Manifestations of Controlling Teaching

The preceding sections explain why controlling teaching persists; these processes become visible through everyday educational interactions. Control may be overt, but it is often expressed through subtle features of language, timing, tone, and routine that progressively narrow learners’ experiences of agency. Examples include premature correction of learners’ reasoning, leading questions asked when answers are already known, advice offered before learners’ perspectives are understood, or directives disguised as choices when compliance is implicitly expected. Repeated over time, such practices can communicate that the safest response is to anticipate expectations, avoid disagreement, and defer to authority rather than openly explore uncertainty.

In high-stakes, hierarchical environments, control may take more overt forms. Learners may experience public correction, exclusion, humiliation, or questioning intended more to expose deficiency than to support reasoning [41]. Because these encounters occur within evaluative relationships, learners may accommodate the behaviour rather than challenge it, while bystanders may hesitate to intervene. Control is therefore sustained not only through individual behaviour, but also through collective silence and the perceived risks of resisting established norms [42].

Control can also be conveyed through body language, relational cues, and the manner in which otherwise legitimate teaching practices are enacted. Public quizzing, comparison among learners, subtle signals of approval and disapproval, or encouragement made contingent on performance may communicate pressure even when educators appear approachable. What matters is whether correction, feedback, structure, and standards promote understanding and ownership or rely on shame, threat, conditional regard, or avoidable pressure.

In other cases, control manifests through educator over-functioning. Educators may take over tasks prematurely, complete learners’ reasoning for them, or restrict meaningful participation because doing so feels more efficient or less risky. Though often justified by workload, time constraints, or patient safety, repeated over-functioning can communicate distrust and deprive learners of opportunities to exercise judgment, experience competence, and assume progressively greater responsibility. Across these manifestations, the defining issue is not how much authority or structure is provided, but whether learners are unnecessarily pressured, bypassed, or deprived of meaningful ownership within the learning process.

## The Costs of Control

For learners, sustained exposure to controlling environments can undermine autonomous motivation, increase psychological need frustration, and shape how learners approach both performance and professional development. Learning may become oriented toward impression management, error avoidance, and compliance rather than exploration, curiosity, and growth. These patterns are especially consequential in clinical education, where learners must be able to disclose uncertainty, seek guidance, and develop progressively greater responsibility.

Educators also bear costs. Autonomy-supportive teaching is associated with greater vitality and satisfaction, whereas controlling motivating styles are associated with diminished enthusiasm and greater emotional exhaustion [31,43]. When control becomes a recurring coping strategy, educators may become caught in cycles of vigilance and responsibility overload that undermine their own psychological needs. These effects may further reinforce control: educator strain can reduce the psychological capacity for patience, perspective-taking, and dialogue, making controlling responses more likely during subsequent periods of pressure.

At the system level, control produces potentially fragile performance: behaviour dependent on surveillance, contingency, and pressure rather than internal commitment. Although such systems may produce immediate compliance and efficiency, they can simultaneously discourage disclosure of uncertainty, impair learning, undermine psychological safety, and contribute to learner disengagement [44]. Ultimately, the defining motivational cost is impaired internalization and integration. Learners may comply with professional expectations without fully endorsing the underlying values, responsibilities, and standards as their own. A system may therefore obtain the appearance of professionalism or competence while weakening the motivational foundations needed to sustain either.

## Implications for Educators, Faculty Development, and Educational Systems

If pressure can travel through educational systems, efforts to reduce controlling teaching must address both how educators respond to demands and where those demands originate. Reflection by individual educators remains important, but it is insufficient when educators work within systems that restrict their own agency, reward certainty, normalize pressure, and leave little room for deliberation or relationally attentive supervision. Asking educators to support learners’ autonomy while providing them with little autonomy in their own work risks reproducing the very dynamic the intervention is intended to change. Sustainable autonomy support therefore requires not only different interpersonal behaviours but also working conditions that protect educators’ own motivation and make need-supportive teaching realistic under pressure.

### Supporting educators’ self-regulation under pressure

At the individual level, controlling teaching may function as a form of regulatory offloading: educators manage their own uncertainty, threatened agency, anxiety, or responsibility by narrowing learners’ agency. Sustaining autonomy support therefore requires the capacity to recognize pressure and reactance without automatically transferring them to learners. This is not simply a matter of applying communication techniques, but of noticing the motivational and emotional processes shaping one’s behaviour in the moment.

Reflective prompts may include:

- What pressure am I experiencing in this moment?
- What is driving my urge to take control?
- Is this level of direction educationally necessary, or is it primarily reducing my own uncertainty?
- How can I provide the structure this situation requires while preserving the learner’s agency?

These questions do not imply that educators should become less directive. Rather, they encourage educators to distinguish necessary clinical direction from attempts to restore their own sense of control. This distinction is especially important when time is limited, stakes are high, or a learner appears disengaged—the circumstances in which controlling responses are most likely to feel justified.

### Clarifying autonomy support, structure, and authority through faculty development

Faculty development provides a critical bridge between individual reflection and organizational change. Many programs teach autonomy-supportive strategies as a list of desirable behaviours, but such approaches may have limited durability when educators are not also helped to understand why control becomes compelling under pressure. Faculty development should therefore move beyond technique alone and help educators distinguish autonomy support from permissiveness, authority from psychological control, and instructional structure from pressure.

Learning should be organized around authentic clinical situations in which these distinctions become difficult to maintain: supervising a struggling learner, conducting an assessment conversation, responding to a patient-safety concern, managing time pressure, or deciding when to intervene directly. Educators can practise acknowledging perspectives, explaining rationales, setting firm expectations, calibrating responsibility, and using direct but informational language without obscuring authority or compromising standards. The aim is not simply to change isolated behaviours, but to develop judgment, self-regulation, and confidence in combining high structure with low control.

Faculty development should also create opportunities for educators to examine the professional beliefs that support controlling practice. If decisiveness is equated with competence, dialogue with weakness, or learner discomfort with educational effectiveness, communication skills alone are unlikely to produce sustained change. Addressing these beliefs makes it possible to reinterpret autonomy support not as a retreat from responsibility, but as a more durable way of exercising it. Faculty development therefore becomes an opportunity to reshape how educators understand pressure, authority, responsibility, and control within clinical teaching.

### Designing need-supportive educational systems

At the cultural and organizational level, institutions should examine the conditions that make controlling teaching seem necessary, effective, or professionally normative. This includes reviewing assessment practices that heighten impression management, administrative communications that rely on compliance without rationale, workload and scheduling arrangements that reduce educators’ capacity for dialogue, and norms that equate authority with certainty or emotional distance.

Educational leaders play a central role in determining whether control is reinforced or reduced. Beyond modelling transparent and autonomy-supportive leadership, they can examine whether assessment systems, administrative processes, and workload expectations inadvertently reward compliance, certainty, or impression management over curiosity, dialogue, and learning. Because many controlling experiences originate outside direct teaching encounters, institutional design itself becomes an important target for intervention.

Leaders can support change by explaining the rationale for requirements, inviting appropriate challenge, creating psychologically safe processes for disclosing uncertainty, and ensuring that accountability systems distinguish between clear standards and controlling enactment. Learners should also be included in examining how policies and routines are experienced, because practices that appear neutral to those with authority may be experienced as pressuring by those subject to evaluation.

These structures should be implemented in ways that support rather than thwart psychological needs. This requires examining not only the formal purpose of educational systems, but also the motivational experience their implementation creates. Documentation should support dialogue rather than replace it; assessment should clarify development rather than encourage accumulation; and accountability should communicate standards without treating pressure as the primary means of achieving them.

Sustainable autonomy-supportive education therefore depends on alignment across levels. Educators require support in regulating themselves under pressure; faculty development must correct misunderstandings about authority and structure; professional cultures must disentangle competence from control; and institutions must reduce avoidable demands that transmit pressure downstream. When these levels are addressed together, autonomy support becomes more than an interpersonal technique—it becomes a property of the educational system.

## Conclusion

Viewing controlling teaching through this lens changes the central question for medical education. Rather than asking only why educators fail to implement autonomy-supportive techniques, it invites examination of why educational systems so consistently make controlling responses appear reasonable, effective, and professionally responsible. This reframing broadens responsibility beyond individual educators without absolving them of agency: educators remain responsible for how they exercise authority, while institutions remain responsible for the conditions under which that authority is enacted.

Control persists not because educators necessarily reject learner-centred values, but because it often works in the short term, restores a sense of predictability or agency, aligns with familiar images of competence, and is reinforced through institutional routines. Pressure imposed from above or experienced from below can therefore travel through educators and become pressure placed on learners. Unless that transmission is recognized and interrupted, autonomy-supportive faculty development may ask educators to absorb demands that the surrounding system continues to reproduce.

Making these dynamics visible allows educators to distinguish necessary direction from unnecessary pressure and enables institutions to identify conditions that repeatedly make control the easiest response. It also reconnects autonomy-supportive education to a central aim of SDT: creating conditions in which learners can internalize professional standards, responsibilities, and values as their own rather than merely complying with external demands.

Sustainable change therefore depends on supporting educators’ own motivation and self-regulation, clarifying the relationship between autonomy support and authority, challenging professional norms that legitimize control, and designing educational systems in which need-supportive practice remains feasible under pressure. Pressure rarely remains where it originates: it is psychologically interpreted by educators, culturally legitimized by professional norms, structurally reproduced by educational systems, and ultimately experienced by learners as controlling teaching. The goal is educational systems in which rigour, accountability, guidance, and responsibility are achieved through support rather than pressure, allowing competence, engagement, and well-being to develop together rather than at one another’s expense.
